# Efficacy and Safety of New *Lactobacilli* Probiotics for Unconstipated Irritable Bowel Syndrome: A Randomized, Double-Blind, Placebo-Controlled Trial

**DOI:** 10.3390/nu11122887

**Published:** 2019-11-27

**Authors:** Joo Hyun Oh, Yeon Sil Jang, Danbee Kang, Dong Kyung Chang, Yang Won Min

**Affiliations:** 1 Department of Medicine, Samsung Medical Center, Sungkyunkwan University School of Medicine, Seoul 06351, Korea; ojh8856@gmail.com (J.H.O.); ys.jang@skku.edu (Y.S.J.); do.chang@samsung.com (D.K.C.); 2 Department of Clinical Research Design and Evaluation, Samsung Advanced Institute for Health Science and Technology, Sungkyunkwan University School of Medicine, Seoul 06351, Korea; cce.smc@samsung.com; 3 Center for Clinical Epidemiology, Samsung Medical Center, Sungkyunkwan University, Seoul 06351, Korea

**Keywords:** irritable bowel syndrome, probiotics, *Lactobacillus*

## Abstract

Irritable bowel syndrome (IBS) is a common and chronic gastrointestinal disorder. Probiotics may have the potential to impact the management of IBS; however, the results of trials are conflicting. This study aimed to investigate whether a mixture of *lactobacilli* probiotics could improve abdominal symptoms in patients with unconstipated IBS. Fifty Vietnamese patients with unconstipated IBS were randomly assigned to either the probiotics or placebo groups. During the intervention, participants took the probiotic supplement, named Foodis Lactobacillus, or placebo capsule once a day. Patients recorded their subject global assessment (SGA) weekly and were assessed with the visual analogue scale (VAS) during the 4-week study period. Patients with SGA score of 2 points or more or a decrease of more than 30% in VAS score were considered responders. Patients who responded weekly for more than 2 of the 4 weeks were considered overall responders. There was no significant difference in demographic characteristics between the groups. Overall responder rates of improvement of global IBS symptoms assessed by SGA score were significantly higher in the probiotics group (80.8%) than in the placebo group (45.8%) (*p* = 0.009). The overall responder rates assessed by VAS score were also higher in the probiotics group (69.2%, 41.7%, *p* = 0.048). There were no adverse events in either group during the study period. Our findings suggest that the new combination of *Lactobacilli* appears to be promising in the relief of abdominal symptoms in Vietnamese patients with unconstipated IBS.

## 1. Introduction

Irritable bowel syndrome (IBS) is a common and chronic gastrointestinal (GI) disorder characterized by abdominal pain and altered bowel habits in the absence of an organic disease. The prevalence of IBS ranges from <5% up to 20% depending on the criteria used [1]. Treating IBS is important because the symptoms cause impairment in health-related quality of life, leading to increased use of health resources and reduced work productivity [2]. While the pathophysiology of IBS is multifactorial involving visceral hypersensitivity, GI motor dysfunction, psychosocial, genetic, and environment factors, as well as intestinal microbiome, attempts to treat IBS have been based on different approaches. There is now increasing evidence linking alterations in the GI microbiota, dysbiosis, and IBS [3]. Gut dysbiosis may be a potential trigger of IBS by increasing intestinal permeability, altering intestinal motility, increasing intestinal sensitivity, and dysregulating the immune system activity [4]. This evidence highlights the potential of modifying gut microbial dysbiosis and bringing therapeutic benefits to IBS patients [5].

Probiotics are live microorganisms which, when administered in adequate amounts, confer a health benefit on the host [6]. Probiotics have been suggested to reduce visceral hypersensitivity or exert anti-inflammatory effects [7,8,9]. Several meta-analyses of randomized controlled trials found probiotics to be more superior to placebo in reducing overall IBS symptoms and abdominal pain [10,11]. Recent studies support that specific bacteria appear to be more efficacious and multi-strain probiotics might be better than single strain because immunological and therapeutic effects of probiotics vary among species [12,13,14]. In addition, gut microbiota differences between ethnicities have been studied and there is the potential to advance approaches aimed at personalized microbial treatment [14,15]. However, much remains to be answered, as which strains and doses are most effective and which regimens of combination work best are still relatively unknown [16].

There are few studies on IBS for Asian people, especially Vietnamese people. The latest research showed that the prevalence of IBS using Rome II criteria was about 10.9% in Vietnam [17]. Of the participants studied, only a minority (5.8%) of those with IBS symptoms reported having visited a physician in the past year for GI complaints [18]. This phenomenon may be due to cultural factors, degree of pain, socioeconomic conditions, and healthcare systems [19,20]. It might be helpful for these patients if they have affordable and accessible medication, such as probiotics. For treating these patients, as a personalized approach, *Lactobacillus salivarius* and *Lactobacillus plantarum* were isolated from the feces of healthy Vietnamese people. In addition, *Lactobacillus paracasei* has been isolated from Kimchi, a traditional Korean food [21,22]. Studies on each of *L. salivarius* [7], *L. plantarum* [23,24,25], and *L. paracase* [26] have been conducted, and these organisms have shown effects on IBS symptoms.

To investigate the probiotic properties of these *Lactobacilli*, an in vitro study was conducted in Korea [22]. The results showed that the three *Lactobacilli* showed promising probiotic activity; excellent resistance to low pH and 0.3% oxgall bile acids; and antioxidant effects, including 2,2-Diphenyl-1-picrylhydrazyl, ABTS stock solution (Sigma, USA), reducing power, and metal chelating (Fe^2+^) activities. In addition, the results showed high binding activity to the mucus layer and heat resistance, which was similar to that of a commercial strain. In our animal models, 40 Wistar rats induced IBS were allocated to *L. paracasei, L. salivarius, L. plantarum*, a mixture of the three, or placebo. The mixture group showed better stool consistency than the control group. The new combination of *Lactobacilli* including *L. paracasei, L. salivarius*, and *L. plantarum* has shown potential utility in IBS without adverse events.

In this study, we investigated the effects of a composite of probiotics on global symptoms and abdominal pain in Vietnamese patients with unconstipated IBS.

## 2. Methods

### 2.1. Study Design, Setting, and Participants

The study was a 4-week, randomized, double-blind, placebo-controlled trial at Samsung Medical Center (SMC), Seoul, Korea. Vietnamese individuals living in Korea aged between 19 and 60 years who met the Rome III criteria [27] for the diagnosis of IBS were eligible to participate. Eligible patients were interviewed using structured questionnaires, visual analogue scale (VAS), and Bristol stool chart (BSC), written in Vietnamese for their symptoms. The exclusion criteria were constipation-predominant IBS (IBS-C), previous history of abdominal surgery except appendectomy and caesarian section, inflammatory bowel disease, and concurrent severe illnesses (cancer, cardiovascular, or pulmonary disease). In addition, patients who had used antipsychotics, antibiotics, and probiotics within 2 weeks were also excluded. Written consent was acquired prior to the study.

**Figure 1:**
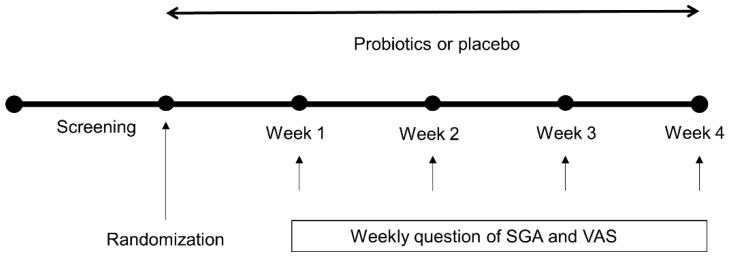
Study design. SGA: Subject global assessment; VAS: Visual analogue score.

This clinical trial comprised a 1-week screening period used to establish the presence of trial entry criteria and to select patients with specified symptoms (Figure 1). During the intervention, participants took either a probiotic mixture capsule which contains one billion colony-forming units or placebo with water or drink once a day (except for acidic juices such as orange juice and soda). Throughout the study, the subjects were not allowed to consume any medications that could influence gut motor or microbiota, including laxatives, antidiarrheal agents, antibiotics, and probiotics. They filled out the weekly questionnaires, which included a 5-point Likert scale of SGA [28] and VAS score [29]. After 4 weeks, an investigator, blinded to the allocation, interviewed the patients with the same structured questionnaires. The study protocol was reviewed and approved by Institutional Review Board at SMC (SMC 2018-01-051-001).

### 2.2. Probiotic Preparation

The probiotic mixture (Foodis Lactobacillus, Ildong Group, Seoul, Korea) contained three strains of the *Lactobacillus* species, *L. paracasei*, *L. salivarius*, and *L. plantarum*. The first *Lactobacillus* was obtained from Kimchi, a traditional Korean fermented food, and the other two species were obtained from feces of healthy Vietnamese. The three strains of *Lactobacillus* were identified by 16S rRNA sequencing. The freeze-dried bacteria were mixed with an excipient and packed into capsules under good manufacturing processing conditions (Foodis, Korea). The proportion of strains in Foodis Lactobacillus was 5:4:1 for *L. salivarius*, *L. plantarum*, and *L*. *paracasei*. These strains were tested for acid tolerance, bile acid tolerance, heat resistance, and mucous layer binding activity, and showed promising probiotic activity. The excipient containing olive oil and pine tree oil was added to the blend of bacteria to achieve the desired dosage concentrations, 1 × 10^9^ CFU/mL. We confirmed stability at each temperature (storing at 15 Celsius, 25 Celsius, and 35 Celsius) for 16 weeks (10 billion maintenance per 2 capsules). Placebo capsules contained the excipient only, which looked identical to the probiotic mixture capsule.

### 2.3. Outcome Measurements

All patients recorded the degree of improvement of their symptoms, assessed as SGA of symptom relief (0 (unchanged), 1 (somewhat relieved), 2 (moderately relieved), 3 (considerably relieved), and 4 (completely relieved)) after 1, 2, 3, and 4 weeks of product consumption. They also recorded abdominal pain scores on a weekly questionnaire as VAS, from 0 (none) to 10 (very severe). The primary outcome was the overall responder rates of adequate relief of IBS-related symptoms. Patients with 2 points or more of the SGA score each week were considered weekly responders. Patients who responded weekly for more than 2 of the 4 weeks were considered overall responders.

The secondary outcome consisted of overall responder rates of abdominal pain reduction (more than 30% decrease in VAS score from the baseline and response for more than 2 of the 4 weeks), weekly responder rates of SGA and VAS, and the changes in 1-, 2-, 3-, 4-week SGA and VAS scores. All the patients were educated to report when they complained of the intervention, such as unintended signs, symptoms, or diseases temporarily without any judgment about causality. Serious adverse events included death, a life-threatening adverse event, inpatient hospitalization or prolongation of existing hospitalization, a persistent or significant incapacity or substantial disruption of the ability to conduct normal life functions, or a congenital anomaly/birth defect.

### 2.4. Statistical Analysis

The sample size was estimated to detect a 35% difference in symptom improvement between the two groups. To achieve 80% power with a two-sided *p*-value <0.05 as significant, it was estimated that at least 31 patients per group were required. A total of 74 patients (37 patients per group) were planned to be randomized in the study, allowing for a 15% dropout rate. Inclusion was halted after the participation of 61 participants because of difficulty in recruiting. Values are expressed as median (interquartile range) or number (%). The random allocation sequence was conducted using a computer-generated, blocked randomization list independent of the research group and with a concealed block. The Student’s *t*-test or Mann–Whitney *U* test was used to compare continuous variables. The group comparison between the placebo and probiotics groups for responders was evaluated with the Chi-square test using SPSS program version 25.0. This study was retrospectively registered with Clinical Research Information Service (CRIS) after enrollment completion (KCT0003831, https://cris.nih.go.kr; date of registration: 23/04/2019).

## 3. Results

### 3.1. Baseline Characteristics

**Figure 2:**
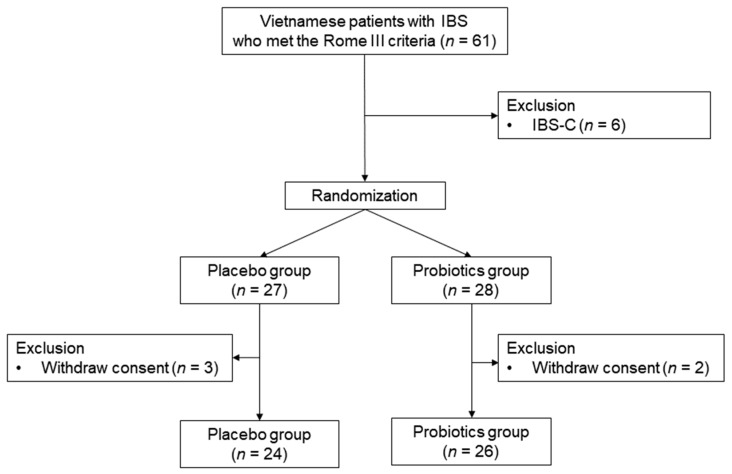
Flowchart of participants. IBS: Irritable bowel syndrome; IBS-C: IBS with predominant constipation.

Sixty-one patients were diagnosed with Rome III criteria of IBS. Three patients from the probiotics group and three from the placebo group were excluded because they had constipation-predominant IBS. A total of 55 patients who met the inclusion criteria were randomized. We further excluded two patients from the probiotics group and three from the placebo group due to the withdrawal of consent. Finally, 26 patients received multi-strain probiotics and 24 received placebo (Figure 2).

The baseline characteristics are shown in Table 1. The two groups were comparable for age, sex, body mass index (BMI), and stool form. At baseline, the symptom score for abdominal pain was similar between the two groups. The median VAS score was 4.0 and 4.0, respectively (Table 1).

### 3.2. Primary Outcome

**Figure 3:**
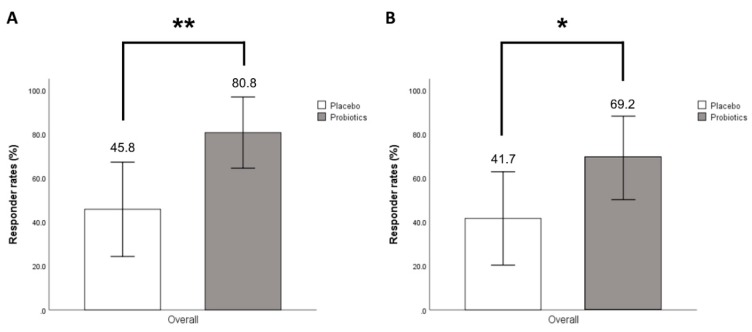
Overall responder rates for improvement of overall IBS symptoms assessed by SGA score (**A**). Overall responder rates for improvement of abdominal pain assessed by VAS score (**B**). * *p* < 0.05, ** *p* < 0.01 compared to placebo. IBS: Irritable bowel syndrome; SGA: Subject global assessment; VAS: Visual analogue score.

There was a significant difference in the proportion of responders between the two groups. Overall responder rates of improvement of overall IBS symptoms assessed by SGA score were significantly higher in the probiotics group than in the placebo group (80.8% vs. 45.8%, *p* = 0.009) (Figure 3A).

### 3.3. Secondary Outcome

**Figure 4:**
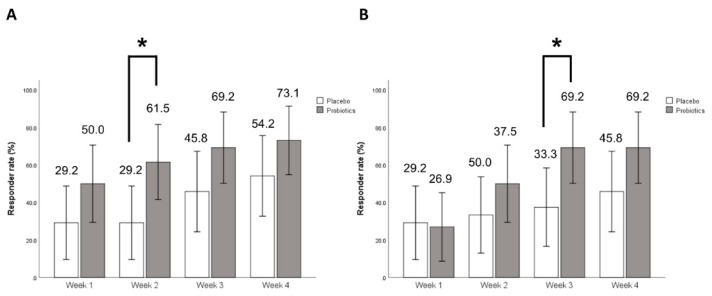
Weekly responder rates for improvement of (**A**) overall IBS symptom assessed by SGA score and (**B**) abdominal pain assessed by VAS score. * *p* < 0.05 compared to placebo. IBS: Irritable bowel syndrome; SGA: Subject global assessment; VAS: Visual analogue score.

Overall responder rates assessed by VAS scores were also significantly higher in the probiotics group than in the placebo group (69.2% vs. 41.7%, *p* = 0.048) (Figure 3B). Serially, both groups demonstrated significant improvements in SGA scores and reductions of VAS scores during the entire treatment period, most notably in the probiotics group. At week 2 in SGA and week 3 in VAS score, the weekly responder rates were distinguishably different. However, the difference in other weekly responder rates did not reach statistical significance (Figure 4).

**Figure 5:**
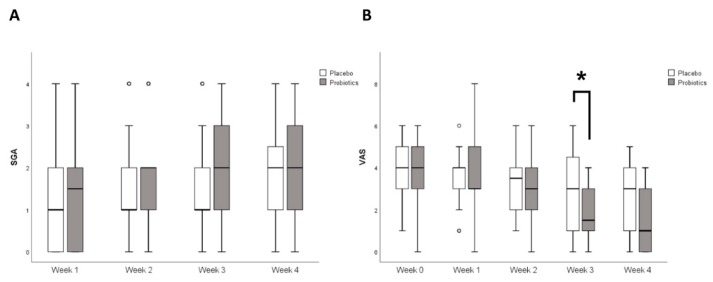
Weekly changes in (**A**) overall IBS symptom assessed by SGA score and (**B**) abdominal pain assessed by VAS score. * *p* < 0.05 compared to placebo. IBS: Irritable bowel syndrome; SGA: Subject global assessment; VAS: Visual analogue score.

Figure 5A,B shows the changes in SGA and VAS scores. After 4 weeks, the mean SGA scores were 2.0 (1.0–2.7) and 2.0 (1.0–3.0) in the placebo and probiotics groups, respectively (*p* = 0.19). Relative to baseline, the mean VAS scores were changed from 4.0 (3.0–5.0) to 3.0 (1.0–4.0) in the placebo group and 4.0 (2.7–5.0) to 1.0 (0.0–3.0) in the probiotics group (*p* = 0.09).

### 3.4. Safety

No adverse events were reported by patients during the 4 weeks.

## 4. Discussion

This study is the first randomized, double-blind, controlled trial of the efficacy of the probiotic composition Foodis Lactobacillus in the treatment of unconstipated IBS in Vietnamese patients. We evaluated overall relief from IBS symptoms as the primary end point because patients with IBS typically complain of complex symptoms, such as abdominal pain, bloating, and altered stool frequency and consistency [28]. The changes in the severity of abdominal pain were also assessed as the secondary outcome. The employment of VAS score is encouraged in treatment trials for IBS by US Food and Drug Administration guidance for IBS. The assessment of stool form and consistency was excluded from the outcomes because the study population included several subtypes of IBS. Through these validated instruments, we demonstrated the therapeutic advantage of probiotics over placebo in IBS symptoms. Although the proportion of weekly responder rates was found to be similar in the fourth week, the Foodis Lactobacillus group showed significantly higher overall responder rates in attenuating overall IBS symptoms and abdominal pain. Moreover, the median VAS scores were maintained better in the probiotics group than in the placebo group throughout the 4-week study period. There were no adverse events in both groups during the study period with dosage concentrations of 1 × 10^10^ colony-forming units per capsule.

A number of studies have been conducted on the effect of probiotics on IBS patients, and some of them have reported favorable outcomes [12,30,31]. On the other hand, several studies failed to show improvement compared to placebo [32,33]. The discrepancy may be due to the heterogeneity among study participants, type of probiotics, usage, as well as methodological differences between trials. Consistent with our study, three meta-analyses concluded that probiotics might have a role in relieving some symptoms of IBS [34,35,36]. There are several reasons why probiotics have a therapeutic benefit in IBS. Their main beneficial effect is acting as a barrier to enteropathogens by adherence and competition with pathogens [37,38]. They also produce some substances that have an antibiotic effect by fermenting undigested carbohydrates and dietary fiber, producing organic acids [39]. Additionally, they may alter the gut microecology, so fewer gases are produced, and that may relieve the symptoms. They increase the efficiency of the immunologic system, particularly intestinal IgA responses, and alleviation of intestinal inflammatory response [40]. These scientific reports suggest the positive effect of probiotics on the host’s health.

Specific probiotic bacteria, *Lactobacillus* species, have been reported to be decreased in stool of IBS patients [41] and emerging evidences suggest that replacement of *Lactobacilli* appears to have beneficial effects on IBS. A multicenter controlled trial of *L. paracasei* showed benefit in global IBS symptoms and reduced stool frequency in patients with diarrhea-predominant IBS [26]. In a 4-week trial, flatulence was significantly improved in the *L. plantarum group* compared with the placebo [25]. In another 8-week trial, abdominal pain and severity scores decreased significantly in the *L. salivarius* group after treatment [7]. These data suggest that the effects are highly strain-specific and it is, therefore, important to choose appropriate species. We tested several *Lactobacilli* and found that *L. paracasei, L. salivarius*, and *L. plantarum* appeared to have beneficial effects in symptomatic improvement in the murine model of IBS. The mixture group showed lower stool consistency scores than the control group (unpublished data). Although the results from the animal model cannot be applied directly to humans, we have tried to use the rationale, which might help to derive positive data.

Molecular and genetic studies allowed the determination of the basics of the beneficial effect of probiotics, involving four mechanisms: Antagonism through the production of antimicrobial substances; competition with pathogens for adhesion to the epithelium and for nutrients; immunomodulation of the host; and inhibition of bacterial toxin production [39]. *L. paracasei* exhibited broad-spectrum antimicrobial activity and was able to inhibit pathogenic bacteria. Damodharan et al. reported *L. paracasei* produced both D- and L-lactate, which could be the reason for the broad-spectrum antimicrobial activity against enteropathogens. *L. paracasei* also showed high inhibition of pathogen adherence by competition and exclusion [42]. *L. salivarius* may influence immune regulation by increased induction of interleukin-10 (IL-10) [43], an anti-inflammatory cytokine, and reduced secretion of proinflammatory cytokines, such as tumor necrosis factor (TNF) alpha, IL-12, and interferon (IFN) gamma [44]. Lastly, the unique ability of *L. plantarum* to catabolize arginine and generate nitric oxide may exert a positive effect on the motility of intestine [45]. The mixture of probiotics may have these therapeutic effects and, therefore, showed positive results. To understand the precise mechanism, evaluation of gut microbial change and visceral sensation is necessary. Further research is needed before making stronger recommendations, perhaps through fecal microbial profiling.

Our data warrant careful interpretation. The study population comprised Vietnamese individuals residing in Korea. There is no evidence that the probiotic effect may vary from population to population, but further studies are needed to investigate whether Vietnamese residents show the same results. The second limitation is the difficulties in recruitment, due to which enrollment was terminated earlier. However, we confirmed significant changes in primary and secondary outcomes, even if the size of the study population was smaller than expected. Lastly, this study did not include a microbiome study, precluding evaluation of the mechanism of action of the probiotics. However, our study may have an advantage in terms of the clues we can provide to the development of effective probiotics, which were extracted from one population with standardized instruments.

## 5. Conclusions

In this randomized double-blinded, placebo-controlled study, the mixture of *Lactobacilli* was effective in the global relief of IBS symptoms, as well as in relieving abdominal pain without significant adverse events. These findings support that probiotics can be considered as a treatment option for patients with unconstipated IBS. Further studies on larger cohorts of patients and with a longer duration of therapy are required.

## Figures and Tables

**Table 1:** Baseline characteristics of participants.

	Placebo (*n* = 24)	Probiotics (*n* = 26)	*p*-Value
Age (year)	33.0 (28.0–44.5)	32.5 (26.5–39.0)	0.28
Female	19 (79.2)	17 (65.4)	0.28
BMI (kg/m^2^)	20.7 (18.3–22.3)	22.2 (19.9–23.6)	0.08
Never smoker	24 (100)	24 (92.3)	0.17
Never drinker	19 (79.2)	20 (76.9)	0.70
Duration of stay in Korea (month)	31.0 (7.0–67.0)	36.0 (14.7–102.0)	0.48
IBS subtypes			0.75
IBS-D	10 (41.7)	11 (42.3)	
IBS-M	6 (25.0)	4 (15.4)	
IBS-U	8 (33.3)	11 (42.3)	
Abdominal pain (VAS)	4.0 (3.0–5.0)	4.0 (2.7–5.0)	0.53
Stool form (BSC)	5.0 (3.2–5.7)	4.5 (4.0–6.0)	0.71

Data are presented as median (interquartile range) or number (%) of patients. BMI: Body mass index; IBS: Irritable bowel syndrome; IBS-D: IBS with predominant diarrhea; IBS-M: IBS with mixed bowel habits; IBS-U: IBS unclassified; VAS: Visual analogue score; BSC: Bristol stool chart.
